# Complexity of Generating Mouse Models to Study the Upper Motor Neurons: Let Us Shift Focus from Mice to Neurons

**DOI:** 10.3390/ijms20163848

**Published:** 2019-08-07

**Authors:** Baris Genc, Oge Gozutok, P. Hande Ozdinler

**Affiliations:** Department of Neurology, Northwestern University, Feinberg School of Medicine, 303 E. Chicago Ave, Chicago, IL 60611, USA

**Keywords:** upper motor neurons, ALS, disease models, reporter lines

## Abstract

Motor neuron circuitry is one of the most elaborate circuitries in our body, which ensures voluntary and skilled movement that requires cognitive input. Therefore, both the cortex and the spinal cord are involved. The cortex has special importance for motor neuron diseases, in which initiation and modulation of voluntary movement is affected. Amyotrophic lateral sclerosis (ALS) is defined by the progressive degeneration of both the upper and lower motor neurons, whereas hereditary spastic paraplegia (HSP) and primary lateral sclerosis (PLS) are characterized mainly by the loss of upper motor neurons. In an effort to reveal the cellular and molecular basis of neuronal degeneration, numerous model systems are generated, and mouse models are no exception. However, there are many different levels of complexities that need to be considered when developing mouse models. Here, we focus our attention to the upper motor neurons, which are one of the most challenging neuron populations to study. Since mice and human differ greatly at a species level, but the cells/neurons in mice and human share many common aspects of cell biology, we offer a solution by focusing our attention to the affected neurons to reveal the complexities of diseases at a cellular level and to improve translational efforts.

## 1. Complexity of the Motor Neuron Circuitry

Movement is one of the most complicated tasks the human body performs. It involves many different neuron and cell types located both in the cerebral cortex and the spinal cord. In addition, the circuitry extends towards muscle, recruiting them as the output of the motor function. Therefore, movement of our muscles is due to an orchestrated and highly controlled set of events that are executed by many different neurons and cells that work together in harmony within the motor neuron circuitry.

The cerebral cortex is the heart of movement as all voluntary movement is initiated in the motor cortex of the brain. The upper motor neurons, which are referred to as corticospinal motor neurons (CSMN) in mice and Betz cells in humans, have a unique importance. They receive many different levels and types of cortical input from long distance projection neurons such as thalamocortical neurons, callosal projection neurons, as well as local circuitry neurons [1,2]. In addition, they are inhibited by an array of different types of inhibitory neurons located both in layer 5 and in layer 2/3 of the cortex. The amount of information the upper motor neurons parallel process within femtoseconds is beyond the capacity of many different neurons in our brain. The upper motor neurons have a long apical dendrite, extending towards the top layers of the brain, and this is the main site of cortical integration. Therefore, the spine density along the apical dendrite and the primary and secondary branches of the apical dendrite, located especially within layer 2/3 and layer 1 is remarkable. Once the upper motor neuron integrates all input coming from many different sources, it may tilt the balance towards generating an action potential, which will be carried long distances towards the spinal cord targets.

Even though upper motor neurons are mostly considered as one neuron type, they can actually be grouped based on their target innervation patterns in the spinal cord. In fact, there is a very precise mapping and target recognition pattern that is developed early postnatally both in mice and humans. The upper motor neurons that innervate the cervical spinal cord are different from the ones that innervate the lumbar spinal cord. The molecular determinants of this targeted precise innervation are beginning to emerge but we still do not know the molecular signature of events that lead to the decision an upper motor neuron makes to exit the corticospinal tract at a precise location and reach out for its targets within the spinal cord. However, this is exceptionally important as it forms the connection between the cortex and the spinal cord and allows the brain to have a direct input to the spinal motor neurons. Even though in humans the cortex–spinal cord connectivity is mostly via direct monosynaptic connections, this is not the case for rodents, especially mice [3]. Many studies have revealed the connectivity patterns between cortex and spinal cord among different species, including cats, dogs, and monkeys [3,4,5]. Interestingly, different species have different types of connectivity patterns and unlike humans they have to rely more on interneurons and interneuron-mediated connections.

The spinal motor neurons also come in many different flavors. They can be grouped based on their size, excitation profile, and the types of muscles they innervate. Therefore, similar to upper motor neurons, not all spinal motor neurons are the same [6]. There is an immense variation among spinal motor neurons as well, and not all degenerate to the same degree and extent in motor neuron diseases, adding one more level of complexity. Interestingly, in some patients, a distinct set of spinal motor neuron may be more vulnerable than others, whereas in other patients, a different group of spinal motor neuron may show initial vulnerability and degeneration. Usually the alpha motor neurons are the ones that are mostly affected and the medium size spiny gamma neurons are spared in ALS patients [6]. The muscle innervation patterns of spinal motor neurons are also of great interest. Not all muscles are innervated with equal distribution of different spinal motor neurons and there is no muscle that is innervated by only one type of spinal motor neuron. There is a plethora of innervation spectrum of muscle fibers by different spinal motor neurons and this variety also adds complexity to the motor neuron circuitry and in part explains the heterogeneity observed in motor neuron disease patients.

Even though the neuronal component is complicated with upper motor neurons, spinal motor neurons, interneurons, and all other excitatory neurons located near and far, there is also a non-neuronal component of the motor neuron circuitry, which includes cells that are not neurons but are as important as neurons for the circuitry function. These cells are mainly astrocytes, microglia, and oligodendrocytes. They have long been considered to have assistive role for proper neuron function. In fact, one of the FDA-approved drugs, Riluzole, acts upon astrocytes and not on motor neurons, to improve the health of the motor neurons. Therefore, improving the health of non-neuronal cells is also critically important for the overall goal of improving the function of motor neuron circuitry. The intricate balance between the neuron–astrocyte interaction and the very many complexities when this interaction is perturbed adds many layers of complexity to the motor neuron circuitry.

Therefore, the motor neuron circuitry with the involvement of numerous different types of neurons in the brain that converge onto upper motor neurons, with non-neuronal cells that play significant roles in synapse formation and maintenance, with the very many different types of spinal motor neurons and the high-level complexity of muscle innervation patterns is one of the most complex systems in our bodies.

## 2. Developing Mouse Models to Study Upper Motor Neurons

The progressive degeneration of upper motor neurons is accepted as one of the major characteristics of neurodegenerative diseases affecting voluntary movement that require cortical input to the motor neuron circuitry. For example, hereditary spastic paraplegia (HSP) is best characterized by the progressive degeneration of upper motor neurons [7]. The disease manifests itself with stiffness in the legs, paralysis, and motor function defects. Primary lateral sclerosis (PLS) is also characterized by upper motor neuron death and CST (corticospinal tract) degeneration. However, in amyotrophic lateral sclerosis (ALS), both the spinal and corticospinal motor neurons progressively degenerate [8,9,10], adding complexity to the disease [11,12].

Discoveries of the genetic causes of diseases that are characterized by the progressive degeneration of upper motor neurons are emerging with a fast speed. Even though a handful of genes were known about ten years ago, today over 60 genes are identified in association with HSP and PLS [13,14,15,16,17], and 147 genes for ALS [18,19,20,21]. When a new mutation is identified in patients, one of the initial modes of action is to generate mouse models that either overexpresses that very human mutation, or a mouse model that lacks the mouse homolog of the gene of interest. In the knockout (KO) model, the overall impact of the protein product that is coded by that mutated gene is investigated on different organs, but mostly on the central nervous system and the motor neuron circuitry. In the overexpression model, the goal is to investigate the impact of the mutated protein on the health and function of cells/neurons, circuitries, and overall survival.

To date, hundreds of different mouse models are generated to investigate different aspects of the disease with the expectation that the mouse model would mimic disease pathology observed in patients. Especially for the motor neuron diseases, the results have been frustrating. With the exception of a few select cases, most of the mouse models did not develop motor function defects; they were able to walk comparable to control cases—albeit some developed gait defects—had life expectancies similar to healthy controls, their brain structure, thickness, and even overall neuron numbers appeared unchanged. This created an unprecedented frustration in the field. Some even chose to blame the mouse as a model system.

The *hSOD1^G93A^* mouse [22] was one of the first models developed and its ability to mimic some of the key features of ALS set the expectation for other mouse models so high that it was expected for a model to display a behavioral outcome. However, we have now come to realize that the *SOD1* model was a special case. SOD1 protein is at the heart of many canonical pathways that are important for the health and function of motor neurons, such as oxidative stress, mitochondrial function, axon transport, and the unfolded protein response, all of which are responsible for motor neuron degeneration. Therefore, in the case of *SOD1*, not only one but many related cellular events and canonical pathways were cumulatively affected, leading to developing an output function that can be measured by behavioral assays and that progressively worsens with age. Unfortunately, not all mouse models were as “lucky”.

Even though the mutated gene in a patient is of great importance for a unique function in motor neurons, its absence or lack of function may not be strong enough to elicit a defect that is easy to measure with behavioral tests in mice. Therefore, the defect remains undetected and it is thus considered “nonexistent” [23,24,25,26,27]. Unfortunately, many of the good models that truly mimic the perturbed biology in affected neurons were considered a “failure” and were put aside without further investigation due to lack of proper outcome measures that could detect true cellular pathology [28,29,30,31]. This has been one of the major limitations in the field.

## 3. Mouse Models Developed with Genetic Linkage to Upper Motor Neuron Diseases

To date, 60 different genes are identified to cause HSP [13,14,15,16,17]. Mouse models of spastic paraplegia with autosomal dominant [32] and autosomal recessive [33] inheritance patterns have recently been reviewed. Here, we focus on motor neuron diseases with upper motor neuron involvement, and availability of mouse models with special emphasis in upper motor neuron defects (Table 1).

Genes that are linked to upper motor neuron dysfunction are emerging. For example, mutations in *ALDH18A1* [198,199], *ERLIN2* [13,16,85,200], *TECPR2* [201], *C12ORF65* [202], *TFG* [203], *ERLIN1* [88], *REEP2* [204], *IBA57* [205], *FARS2* [206,207], *ZFYVE27* (*PROTRUDIN*) [208], *PLSA1* [209,210] genes were detected in a broad spectrum of patients with upper motor neuron involvement. However, mouse models have not yet been generated.

Interestingly, some mouse models were generated even prior to the identification of genes in relation to upper motor neuron dysfunction (Table 1, highlighted green). These models thus were used to investigate pathologies that are not related to motor neuron diseases, such as detection of protein levels in the liver and kidney [37], observation of bile acid synthetic enzyme expression [36], investigating of wavy hair phenotype [57], lung injury [91], understanding prolonged bleeding times [94], colitis [92], investigating vascular and immune abnormalities [211], and diabetic nephropathy [93]. The cortical component was not investigated in detail, even though some of them, such as *Gjc2* showed a motor function defect and a phenotype suggesting upper motor neuron involvement. Therefore, we suggest that these mouse models may offer insight to further reveal the underlying causes of upper motor neuron degeneration.

Upon identification of genes that lead to motor neuron diseases when mutated, a number of mouse models have been generated using different mouse genetics. One of the first lines of investigation is performed via behavioral analyses, using a battery of tests including rotarod, beam-walking, clasping response test, extension reflex test, wire hang test, horizontal pole test, treadmill walking, and measuring gait angles and step sequences. Most of the mouse models failed to display a motor dysfunction and were comparable to control groups, (i.e., *Cy7b1, L1cam, Ddhd1, Kif1a, Fa2h, Nt5c2, Gba2 Ap4, Ampd2, Entpd1, Atl1, Spast, Kiaa0196, Rtn2, Reep1* mouse models) (Table 1, highlighted in blue).

Interestingly, a small percentage of mouse models did indeed display gait abnormalities, motor function defects that emerge at later ages, and defects that suggest upper motor neuron involvement (i.e., *Paraplegin*, *Spatacsin*, *Zfyve26*, *Spartin*, *Maspardin*, *B4galnt1*, *Pnpla6*, *Gjc2*, *Ap5*, *Ddhd2*, *Mag*, *Capn1*, *Atp13a2*, *Uchl1*, *Nipa1*, *Sspd1*, *Bscl2*, *Lsc33a1*, *Cpt1c* mouse models, Table 1). Because *Kif5a* null mutants die immediately after birth, a *Synapsin*-promoter Cre-recombinase transgene was used for selective inactivation of *Kif5a* in neurons postnatally. Three fourths of mutant mice exhibited seizures and death at around 3 weeks of age. Nuclear area was found significantly smaller in *Kif5a*^−/^^−^ spinal motor neurons in comparison to *Kif5a*^+/+^ controls. *Kif5a*^−/^^−^ spinal motor neurons, as identified by morphology by anti-Islet, anti-Chat, anti-Map2, and anti-phospho-tau staining, showed reduced survival [24]. *Paraplegin*-deficient mice were affected by a distal axonopathy of spinal and peripheral axons, characterized by axonal swelling and degeneration. Mitochondrial morphologic abnormalities occurred in synaptic terminals and in distal regions of axons long before the first signs of swelling, and correlated with onset of motor impairment and degeneration. Axonal swellings occurred through massive accumulation of organelles and neurofilaments, suggesting impairment of anterograde axonal transport, while retrograde axonal transport was delayed in symptomatic mice [43]. In addition, an early-onset severe neurologic phenotype in *Spg7*-null/*Afg3l2*^+/−^ mice characterized by loss of balance, tremor, and ataxia were detected. These mice displayed acceleration and worsening of the axonopathy as observed in *Spg7*-null mice [45]. *Seipin* KO mice displayed anxiety and depression-like symptoms. Neuron-specific *Seipin* KO mice also showed reduced mRNA and protein levels of *Pparg* in hippocampus and cortex [160]. Investig.igation of age-related motor dysfunction in *Atp13a2* null model revealed gliosis, accumulation of ubiquitinated protein aggregates, lipofuscin, and endolysosomal abnormalities in cortex [113]. *Ddhd2*^−/−^ mice had shorter stride lengths in gait measurement assays and this locomotor defect was observed both front and hind paws. In addition, it showed significant reduction in rearing behavior and rotarod balance was shortened [87]. The *Spartin* mouse model generated by targeted disruption of *Spg20* gene shows significant gait phenotype and, interestingly, cerebral cortical neurons cultured from *Spg20^−/−^* mice exhibited increased axonal branching [53].

To date, four different mouse models for *Spast* have been generated. To understand the involvement of Spastin in synapse elimination and microtubule destabilization, a *Spastin* knock out mouse was generated via the “knockout-first” approach. In this mouse, *Spastin* deletion caused no obvious phenotype in young animals [138,140]. In the *Spast^KO^* mice, exons 5–7 of the *Spast* gene were deleted, introducing an early stop codon. Homozygous mutant mice developed a mild and late onset motor defects at 22 months [137,140]. Axonal swellings, impaired microtubule disassembly and reduced microtubule plus ends were identified, only in homozygous mutant mice. Deletion of mouse *Spast* gene, generating a premature stop codon, is responsible for axonal degeneration, restricted to the central nervous system, leading to late and mild motor defect [140]. The second *Spast^KO^* model was generated by deletion of exon 7 of the *Spast* gene [138]. Similar to the previous model, axon swellings were present and homozygous mutant mice developed slight gait abnormalities, detected as early as 7 months. In both models, anterograde axonal transport of mitochondria was prominently impaired, while retrograde transport remained relatively intact. The third model is the *Spast^N386K^* knock in model, in which N386K was introduced into the endogenous *Spast* locus within its AAA domain [212]. Only homozygous mutant mice showed abnormalities in gait parameters and axonal swelling were present in cultured cortical neurons. The fourth mouse model is the *SPAST^C448Y^* transgenic mice, which is generated by the insertion of the human full-length *SPAST* harboring C448Y into the Rosa26 locus [139]. Both heterozygous and homozygous mice show severe gait impairment, and male mice display a more severe phenotype.

*Zfyve26* deficient mice generated by deleting exon 15 of the *Zfyve26* gene. Young *Zyfve26* KO mice did not show any obvious abnormalities or altered body weight compared to wild type littermates up to 8 months of age. At 16 months of age, the body weight of the knock out mice was reduced. At 12 months of age, KO animals showed progressive gait disorder and motor deficits. These are quantified by measuring foot base angle at toe off positions of the hind paws. Disruption of *Zfyve26* caused severe neuron loss in the motor cortex and cerebellum [47].

The *spatacsin* mouse model was generated by disruption of *Spg11* gene in mice via inserting stop codons in exon 32. It developed early-onset motor impairment and cognitive deficits. The behavioral deficits were associated with progressive brain atrophy with the loss of neurons in the primary motor cortex, cerebellum, and hippocampus as well as accumulation of dystrophic axons in the corticospinal tract. Spinal motor neurons also degenerate [47].

*Cpt1c*-deficient mice develop early onset of progressive motor disturbances, including impaired gait and coordination, severe muscle weakness and reduced locomotor activity. Cerebellar, striatum, and motor cortex extracts from *Cpt1c*-KO mice show reduced levels of ceramide and its derivative, sphingosine, mainly during fasting state, compared to wild type mice. Mice were assessed neurologically and behaviorally, and results showed impaired coordination, hypoactivity, and reduced muscle strength. *Cpt1c* KO mice also showed reduced levels of ceramide and sphingosine in the cerebellum, striatum, and motor cortex detected by Western blot analysis [170].

Heterozygous mice for a KO allele of the *Hspd1* gene, encoding *Hsp60* (*Sspd1*), demonstrate that *Hspd1* haploinsufficiency is sufficient to cause a late disease onset in mice. These mice were tested behaviorally and analyzed for mitochondrial ATP production. They displayed a marked and progressive deterioration in performance of all motor tests performed compared to wild type littermate control mice [155]. A transgenic mouse in which exon 2 of *Reep1* was removed and immunoblot studies with an antibody recognizing a C-terminal epitope showed that full-length Reep1 protein is absent in mice homozygous for the mutant transgene [213]. Behavioral examination of *Reep1*-null mice that were less than one-year of age did not reveal any obvious motor deficits. Older mice showed changes in hind limb function but more rigorous quantitative analysis revealed the onset of motor deficits at an earlier time point; a change in the foot/base angle during ambulation of 4 to 5-month-old *Reep1* KO mice. Evaluation of 13-month old *Reep1*^−/^^−^ mice did not reveal decrease in the motor cortex; however, ultrastructural studies of 7.5-month-old mice uncovered axonal deficits in the corticospinal tract. Careful EM studies of layer 5 pyramidal cells in the motor cortex of one-year-old mice showed a Reep1 dose-dependent increase in the average length and decrease in the number of individual ER structures. Loss of *Reep1* decreases ER curvature, resulting in a reduction in the apparent number but an increase in the length of ER tubules [163].

Quantitative dendritic tree analysis was performed on layer 3 and layer 5 pyramidal neurons in the primary motor cortex of *B4galnt1* null mice. The layer 5 neurons were observed as more mature, having larger cell bodies and more branched dendritic trunks at P3 [23]. However, Calbindin immunohistochemistry was performed to quantify Purkinje cell numbers in *Ap5* mouse model [84], and the motor cortex was not investigated in detail. These mice accumulate autofluorescent material in neurons and develop late onset progressive gait abnormalities, recapitulating the human phenotype. In agreement with a role of *Ap5* for the retrieval from late endosomes to the *trans*-Golgi network, several Golgi-related proteins were enriched in lysosomal fractions of KO mouse embryonic fibroblasts.

Since upper motor neurons make up of less than one percent of the motor cortex neurons and cells, it is challenging to reveal the extent of their degeneration, and thus most of these studies concluded no prominent upper motor neuron loss. (i.e., *B4galnt1*, *Pnpla6*, *Kif5a*, *Bscl2* mouse models). One example is the *Alsin* KO mice, which was developed after mutations in the *ALSIN* gene were detected in juvenile ALS cases [214]. Four different groups generated mouse models for *Alsin*, but the mice did not have a profound motor function defect, even though it displayed gait abnormalities when aged. Immunocytochemistry analysis using neuronal markers such as Neun, did not reveal neuronal loss, and thus it was concluded at the time that the cortical neurons were unaltered in these mouse models. With the identification of molecular markers that are more specific to upper motor neurons in the motor cortex, such as Ctip2 [215], more cell-type-specific degeneration patterns of upper motor neurons were investigated. For example, in *Spatacsin* [48] and *Uchl1* [120] mouse models, Ctip2 immunocytochemistry suggested a progressive degeneration of upper motor neurons with age, albeit different mouse models displayed different rates and extent of upper motor loss. Similarly, in the *Alsin* KO mice, the Ctip2 immunocytochemistry revealed upper motor neuron loss that could not be detected by Neun expression [28]. These studies further suggested the importance of visualization and direct cellular assessment of upper motor neurons for revealing the underlying mechanisms that are responsible for their vulnerability and degeneration.

## 4. Visualization of CSMN

**Figure 1:**
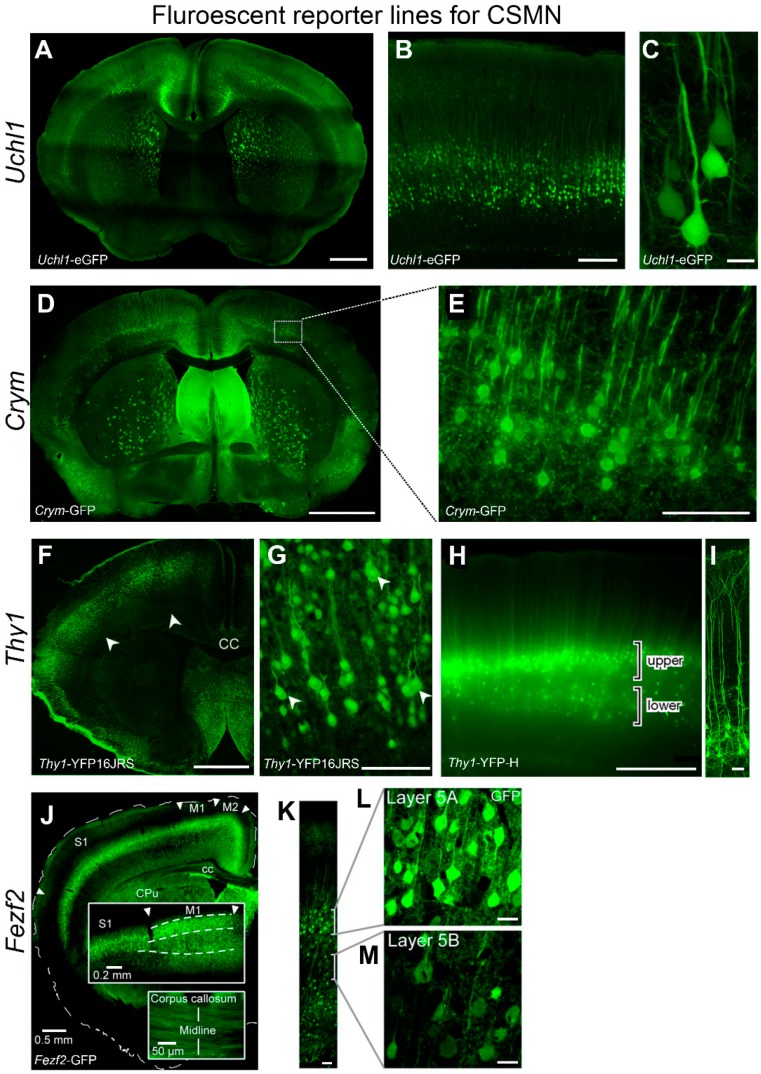
Reporter mouse lines available for visualizing CSMN. (**A**–**C**) *Uchl1*-eGFP reporter mouse line. Low magnification image of coronal section through primary motor cortex (**A**), GFP labeled neurons in layer 5A and 5B of primary motor cortex (**B**), and high magnification image of CSMN (**C**). (**D**,**E**) *Crym*-GFP reporter mouse line. Low magnification image of coronal section through primary motor cortex (**D**), GFP labeled neurons in layer 5 of primary motor cortex (**E**). (**F**–**I**) *Thy1*-YFP reporter mouse line. Low magnification image of coronal section through primary motor cortex (**F**), and YFP labeled neurons in layer 5 of primary motor cortex (**G**) in the *Thy1*-YFP16JRS mouse. Low magnification image of a section through primary motor cortex (**H**), and YFP labeled neurons in layer 5 of primary motor cortex (**I**) in the *Thy1*-YFP-H mouse. (**J**–**M**). *Fezf2*-GFP reporter mouse line. Low magnification image of coronal section through primary motor cortex (**J**), GFP labeled neurons in layer 5A and 5B of primary motor cortex (**K**), and high magnification image of layer 5A (**L**) and layer 5B (**M**). Scale bars: 1 mm in (A), 250 μm in (B), 20 μm in (C), 1 mm in (D), 100 μm in (E), 1 mm in (F), 100 μm in (G), 500 μm in (H), 50 μm in (I), 0.5 mm in (J), 50 μm in (K), 20 μm in (L–M).

Recently, numerous novel techniques and approaches have been developed to help identify and visualize CSMN within the complex structure of the cerebral cortex. Retrograde labeling surgery, AAV-mediated gene delivery, and novel reporter lines now have the potential to change the future of CSMN investigations [2,120,216,217]. Reporter lines have been very informative on marking the cells and neurons of interest with fluorescence. To date, numerous reporter lines are generated which genetically labels upper motor neurons with fluorescence (Figure 1). Even though the labeling may not be cell-type specific in all of the reporter lines, presence of fluorescence allows detailed cellular analyses. *Mu-crystallin* (*Crym*)-GFP reporter is a high-fidelity marker of the CST [218]. CST labeling with *Crym*-GFP is ten times more efficient compared with BDA; however, low-level expression requires significant amplification of the GFP signal using immunofluorescence techniques. *Thy1*-YFP mice have extensively been used to study motor systems, not only SMN in mouse models of ALS [219,220], but also CSMN [221,222,223] and their axons in spinal cord injury [224] and an ALS mouse model [225]. *Fezf2*-GFP labels a heterogenous population of neurons that include corticospinal projection neurons and corticothalamic projection neurons in layer 5A and crossed corticostriatal projection neurons and crossed-corticocortical projection neurons in layer 5B of the mature motor cortex [226].

### UCHL1 Offers a Unique Opportunity to Study Upper Motor Neuron Biology

*Uchl1*-eGFP mice in which the *Uchl1* gene promoter is used to drive eGFP expression has been invaluable in selectively labeling CSMN in mice [227]. CSMN identity of eGFP+ neurons was confirmed by retrograde labeling, molecular marker expression profile, electrophysiology, cortical circuit mapping, and mouse genetics studies. CSMN in the motor cortex and their projections were genetically and stably labeled by GFP expression from P0 to P800. In the spinal cord, almost all ChAT^+^ SMN were eGFP^+^ at birth but, by P30 eGFP expression, became mostly restricted to a mixture of small α- and γ-SMN that are resistant to generation in motor neuron diseases, such as ALS. Crossing this reporter mouse with *hSOD1^G93A^* ALS mouse model [22] generated *hSOD1^G93A^*-UeGFP mice, which allowed detailed study of CSMN health with respect to mSOD1 mediated ALS. We observed a progressive degeneration of eGFP^+^ CSMN, as previously reported [225], with apical dendrite vacuolation and presence of autophagosomes, suggesting an ongoing intrinsic cellular degeneration.

Ubiquitin C-terminal hydrolase ligase 1 (UCHL1) is one of the most abundant proteins in the brain [228,229]. It is an important component of the ubiquitin–proteasome system (UPS) and can either add or remove ubiquitin to polyubiquitin chains [228,229,230]. Inhibition of Uchl1 results in a 50% reduction of free ubiquitin in vitro [231,232]. Absence of Uchl1 function in vivo leads to accumulation of ubiquitinylated proteins in motor cortex and increased ER stress in CSMN [120], enhanced neuronal protein synthesis and proteasomal protein degradation, with endoplasmic reticulum stress, and energy depletion, leading to proteasomal impairment and an accumulation of nondegraded ubiquitinated protein [124]. Increased protein turnover is associated with enhanced mTORC1 activity restricted to the postnatal period in *Uchl1*-deficient brains [124]. Uchl1 also regulates the balance between mTOR complexes by disrupting mTORC1 and promoting mTORC2 assembly [233]. Overexpression of Uchl1, on the other hand, leads to cancer [234,235]. The active site of Uchl1 required for its hydrolase activity contains a triad of Cys^90^, His^161^, and Asp^176^ [228,236]. Catalytically inactive *Uchl1^TgC90A^* mice indicate that its catalytic activity is essential for the oncogenic effects of Uchl1 in mice [233].

Mutations in the *UCHL1* gene cause autosomal recessive spastic paraplegia-79 (SPG79) (MIM Number: #615491) [14,119,237,238]. The UCHL1^GLU7ALA^ missense mutation identified in a Turkish family lies within the ubiquitin binding domain of UCHL1 protein and leads to near complete loss of hydrolase function [119]. All three siblings homozygous for the mutation have spasticity with upper motor neuron dysfunction, accompanied by early onset blindness, cerebellar ataxia, nystagmus, and dorsal column dysfunction. Two other missense mutations in the *UCHL1* gene were identified in a Norwegian family [238]. Three siblings with compound heterozygous mutations *UCHL1^ARG178GLN^* and *UCHL1^ALA216ASP^* developed spasticity and ataxia following child onset blindness. Whereas *UCHL1^ALA216ASP^* was reported to be insoluble and therefore nonfunctional, *UCHL1^ARG178GLN^* mutation affects a rate-controlling residue in catalysis leading to a four-fold increase in hydrolytic activity of the UCHL1 protein. Recently, a third family from India was reported with two siblings carrying a deleterious homozygous splice-site variant predicted to cause splicing aberrations [237]. Both siblings have spasticity and child onset optic atrophy. Clinical features of all eight patients from three families are comparable and include spasticity, indicating upper motor neuron involvement.

There are several mouse models of *Uchl1* available, some have spontaneous deletions within the *Uchl1* region, and others with targeted deletions to generate *Uchl1* KO mice. *Uchl1^nm3419^* mice arose as a spontaneous deletion in the BL6 colony of Jackson laboratories displaying motor defects, and later were identified to carry a 795 base-pair intragenic deletion that results in the removal of 24 base-pairs of exon 6 and 771 base-pairs of intron 6 [122]. The *Uchl1^nm3419^* mice, which lack all Uchl1 function display motor function defects as revealed by rotarod and grip test analysis [120]. By using known molecular markers of CSMN such as Ctip2 [215] or using retrograde labeling surgery [2], we were able to show progressive CSMN loss and cellular degeneration that is revealed by vacuolated apical dendrites, spine loss, and increased ER stress [120]. Interestingly, the SMN are also affected, even though they do not undergo massive cell loss as observed in CSMN. Muscular atrophy and distal degeneration of SMN axons is observed in which the neuromuscular junctions (NMJ) are denervated and lose their integrity [128].

Gracile axonal dystrophy (Gad) mice also have a spontaneous deletion but include the exons 7 and 8 encoding a truncated Uchl1 lacking a segment of 42 amino acids and containing a catalytic residue instead [121,126]. Gad mice develop sensory and motor ataxia, hindlimb paralysis, and degeneration of distal motor axons [121,129,239]. Unfortunately, to our knowledge, the motor cortex of these mice has never been studied and the CSMN degeneration remains to be investigated.

A *Uchl1* KO mouse model has been generated with targeted deletion of a region containing exons 6 through 8 and the first six base pairs of exon 9 of *Uchl1* [125]. Similar to spontaneous deletions of *Uchl1*, these animals also develop an ataxic phenotype with progressive motor defects leading to paralysis and degeneration of motor axons at the NMJs. Motor cortex and CSMN involvement has not been investigated. Recently, a floxed *Uchl1* mouse line has been generated in which the exons 1–3 of the *Uchl1* gene are flanked by loxP sites [124]. By crossing the floxed *Uchl1* mice with constitutive deleter Cre mice, constitutive *Uchl1* deficient (*Uchl1^d/d^*) mice have been generated, which developed progressive motor defects similar to other *Uchl1* mouse models, including reduced performance in accelerating rotarod and open field tests and reduced forelimb strength. In whole brain lysates, levels of polyubiquitinated proteins were drastically decreased in 3-week-old *Uchl1^d/d^* mice and remained lower compared with *Uchl1^+/+^* mice. Uchl1 deficiency resulted in decreased levels of polyubiquitinated proteins in juvenile mice, followed by an abnormal accumulation of polyubiquitinated proteins in old adult mice, resulting in an upregulation of proteasomal levels and ER stress. Data suggests *Uchl1* is involved in regulation of protein synthesis in neurons before the first symptoms are observed and Uchl1 deficiency enhances mTOR activation. Although increased ER stress in the absence of Uchl1 is in line with our findings in the *Uchl1^nm3419^* mice, CSMN loss and degeneration has not been investigated in this mouse model.

Although several *Uchl1* mouse models exist, CSMN degeneration has been extensively studied mainly in the *Uchl1^nm3419^* mice [120]. Thus, the *Uchl1^nm3419^* mouse model remains the best characterized motor neuron disease model in terms of upper motor neuron involvement [120]. The *Uchl1* null mice reveals very important knowledge on the cellular events that are responsible for upper motor neuron degeneration.

## 5. Shifting Focus from Mice to Neurons Generates Translational Outcomes

**Figure 2:**
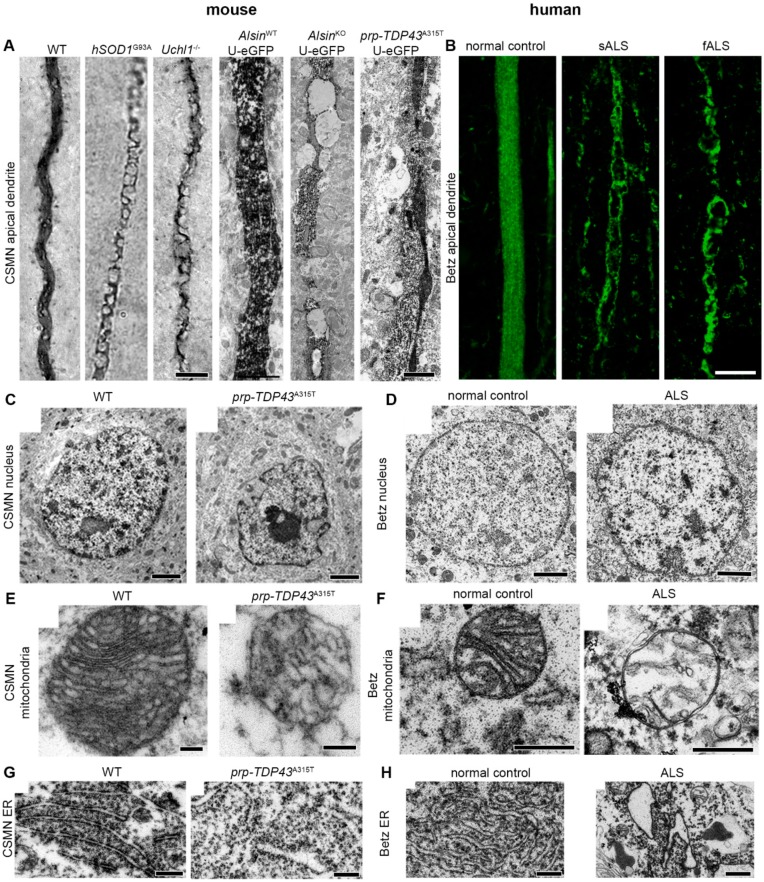
Betz cell pathology is similar between mouse and human at a cellular level. (**A**) Apical dendrites of CSMN displaying vacuoles in brains of various mouse models of motor neuron disease. (**B**) Apical dendrites of Betz cells displaying vacuoles in brains of patients with sporadic or familial ALS. (**C**–**H**) Electron microscope images showing pathology of various organelles in CSMN of *prp*-*TDP43*^A315T^ mouse and Betz cells of ALS patients. Observe similar nuclear membrane defects (**C**,**D**), mitochondria defects (**E**,**F**), and endoplasmic reticulum defects (**G**,**H**). Scale bars: 5 μm (brightfield, left), and 1 μm (E.M., right) in (A), 10 μm in (B), 2 μm in (C), 500 nm in (D), 200 nm in (E), 500 nm in (F), 1 μm in (G–H).

Compounds that extended the life-span of mouse models failed to improve the life-span of patients and this was considered as a “failure” for translational efforts [240,241,242,243,244,245,246,247,248]. However, mouse models remained one of the most essential components of preclinical investigations [248]. Therefore, it is important to choose the model that best represents the biology of interest. In addition, being able to visualize and cellularly assess the direct response of diseased neurons to treatment is of great importance. Many mouse neurons are similar and almost identical to the neurons in humans. Their birth, differentiation, maturation, target recognition, and circuitry integration patterns appear to be very similar. We also found that the cellular basis of upper motor neuron degeneration is almost identical between mice and humans. For example, the apical dendrite degeneration detected in many different mouse models of ALS was also detected in a broad spectrum of ALS patients, including sALS, fALS, and ALS/FTLD cases. The nucleocytoplasmic transport defects reporter in patients with TDP-43 pathology was also observed in CSMN of mice with Tdp-43 pathology. The mitochondrial defects, problems with ER were all comparable and similar between two species, when the vulnerable neurons were investigated at a cellular level (Figure 2) [249,250]. This very close correlation and direct recapitulation of cellular events in two different species led us to believe that the translation will be at a cellular level and that focusing our attention to the vulnerable and diseased neurons of well-defined mouse models of the diseases will inform us on the vulnerable and diseased neurons of patients, and this information forms the foundation for all translational research [251,252].

Taking advantage of the existing mouse models for the numerous genes that are known to cause motor neurons disease, we now can cross them with *Uchl1*-eGFP mice or other well-defined GFP reporter lines, retrogradely label CSMN surgically using fluorescent markers or AAV containing GFP expression vectors to study them at a cellular level. This will not only allow us to visualize the CSMN in these animal models, but also to isolate and purify them for further omics approaches that would shed light on the mechanism of disease onset and progression.

In some cases, more detailed functional assays are required to assess the importance of the identified gene or pathway in upper motor neuron health and survival. Crossing genes of interest that are floxed with *Rbp4*^cre^ mice that express Cre recombinase, and the control of the *Rbp4* promoter targeting subcerebral projection neurons that lie in layer 5, including the CSMN in the primary motor cortex, [253,254,255,256] would allow us to investigate the impact of genetic alterations selectively in CSMN, without affecting other cells and neurons in the circuitry. This approach is also very powerful to determine whether genes of interest would be potential drug targets in patients.

In neurodegenerative diseases, not all neurons are affected to the same extent. While some show initial vulnerability, many others remain unaffected until the end-stages of the diseases. Therefore, it is critically important to understand why a particular neuron population begins to suffer much earlier than others. This line of investigation is only possible when we bring clarity and transparency to cellular analyses. Since the cerebral cortex is very heterogenous and complex, this has been challenging. However, thanks to current developments, it is now possible to visualize and study distinct neuron populations with cellular precision that was not possible before. Such studies reveal very strong correlation between the upper motor neurons in mice and humans, reinforcing the idea that the upper motor neurons in two different species are almost identical at a cellular level. Focusing our attention on the neurons that display early vulnerability and undergo progressive degeneration in diseases will be translational and transformative. At the end of the day, our goal is not to cure the mice, but to improve the health of the neurons that degenerate. Shifting our focus from mice to neurons will help generate the translational information needed for building effective treatment strategies for patients. We now have the appropriate tools to shed light onto upper motor neurons and to build effective treatment strategies for diseases in which voluntary movement is affected.

## Figures and Tables

**Table 1:** Genes for motor neuron disease with upper motor neuron involvement, the mouse models generated, and the investigation of the cortical component of motor neuron circuitry.

Disease	Gene	Mouse Model Available	Motor Cortex Involvement
SPG5A	*CYP7B1* [34,35]	Y [36,37]	
SPG7	*PARAPLEGIN* [38,39,40,41,42]	Y [43,44,45]	
SPG11	*SPATACSIN* [46]	Y [47,48]	Y [47,48]
SPG15	*ZFYVE26* (*SPASTIZIN*) [49]	Y [50]	Y [50]
SPG20	*SPARTIN* [51,52]	Y [53]	
SPG21	*MASPARDIN* [54]	Y [25]	N [25]
SPG26	*B4GALNT1* (*GM1, GALNACT*) [55]	Y [23]	N [23]
SPG28	*DDHD1* (*PAPLA*) [56]	Y [57,58]	
SPG30	*KIF1A* [59]	Y [60,61,62]	
SPG35	*FA2H* [63]	Y [64,65]	
SPG39	*PNPLA6* (*NTE*) [66]	Y [26,67]	N [26]
SPG44	*GJC2* (*CX47*) [68]	Y [69,70,71,72]	
SPG45	*NT5C2* [73]	Y [74]	
SPG46	*GBA2* [75,76]	Y [77,78,79,80]	
SPG47	*AP-4* [81]	Y [82]	
SPG48	*KIAA0415* (*AP-5Z1*) [83]	Y [84]	Y [84]
SPG54	*DDHD2* (*KIAA0725P*, *IPLA1γ*) [85]	Y [86,87]	
SPG63	*AMPD2* [88]	Y [89,90,91]	
SPG64	*ENTPD1* (*CD39*) [88]	Y [92,93,94]	
SPG75	*MAG* [88,95]	Y [96,97,98,99,100,101,102,103]	
SPG76	*CAPN1* [104]	Y [105,106,107,108]	
SPG78	*ATP13A2* [109,110]	Y [111,112,113,114,115,116,117,118]	
SPG79	*UCHL1* [119]	Y [120,121,122,123,124,125,126,127]	Y [120,124,125,128,129]
SPG3A	*ATL1* [130,131,132]	Y [133,134]	
SPG4	*SPAST* [135,136]	Y [137,138,139,140]	Y [140]
SPG6	*NIPA1* (*CXFIP1*) [141]	Y [142]	
SPG8	*KIAA0196* (*WASH C5, STRUMPELLIN, RTSC1*) [143,144,145]	Y [146]	
SPG10	*KIF5A* [147]	Y [24,148]	N [24]
SPG12	*RTN2* (*NSPL1*) [149]	Y [150]	
SPG13	*SSPD1* (*HSP60*, *HSPD1*) [151]	Y [152,153,154,155]	Y [153,155]
SPG17	*BSCL2/SEIPIN* [156,157]	Y [27,158,159,160]	N [27]
SPG31	*REEP1* [161,162]	Y [163,164,165]	Y [163,165]
SPG42	*SLC33A1* [166,167]	Y [168]	
SPG73	*CPT1C* [169]	Y [170,171]	Y [170]
SPG1	*L1CAM* [172]	Y [173,174,175]	Y [173,174]
SPG2	*PLP1* [176,177,178,179]	Y [176,180,181,182,183,184,185,186,187]	
PLS	*ALS2* [188,189,190,191,192]	Y [28,193,194,195,196,197]	Y [28]

## Abbreviations

ALS	Amyotrophic Lateral Sclerosis
PLS	Primary Lateral Sclerosis
HSP	Hereditary Spastic Paraplegia
CSMN	corticospinal motor neuron(s)
CST	corticospinal tract
EM	electron microscopy
ER	endoplasmic reticulum
UCHL1	ubiquitin C-terminal hydrolase ligase 1
UMN	upper motor neuron
GFP	green fluorescent protein
KO	green fluorescent protein
